# RESET-APP: ein App-basiertes Angebot zur Selbstregulation für Schüler*innen der Sekundarstufe I – Akzeptanz und Nutzungsverhalten

**DOI:** 10.1007/s11553-022-00952-2

**Published:** 2022-05-20

**Authors:** Katharina Bundscherer-Meierhofer, Margarete Rauch, Thomas H. Loew, Beate Leinberger

**Affiliations:** Abteilung für Psychosomatische Medizin, Universitätsklinikum Regensburg, Franz-Josef-Strauß-Allee 11, 93053 Regensburg, Deutschland

**Keywords:** App-basiertes Training, Selbststabilisierung, Jugendalter, Motivation, Stressmanagement, Digital technology, Self-stabilisation, Adolescents, Motivation, Stressmanagement

## Abstract

**Hintergrund:**

Die COVID-19-Pandemie („coronavirus disease 2019“) hat immense Auswirkungen auf die psychische Gesundheit. Kinder und Jugendliche gelten hierbei als besonders vulnerabel. Deshalb sind gerade für sie Angebote zur Gesundheitsförderung und Prävention wichtig. Gesundheitsförderung sollte motivierend gestaltet werden, um für diese Altersgruppe attraktiv zu sein. Ziel dieser Arbeit war zu untersuchen, ob Jugendliche mithilfe von innovativen digitalen Formaten erreicht werden können.

**Methodik:**

Es wurde ein App-basiertes Training zur Förderung der Selbstregulation für die Sekundarstufe I konzipiert und Jugendlichen der Sekundarstufe I im Herbst 2020 angeboten. Hier wurde u. a. die Art der Motivation zur Teilnahme am Training abgefragt. Zudem konnten die Schüler*innen die Attraktivität des Trainings abschließend bewerten.

**Ergebnis:**

Von den registrierten Schüler*innen (*n* = 91) absolvierten 39,56 % das komplette Training. 40,91 % der Schüler*innen, die das Training vollständig absolviert haben, gaben an, dass das Training „sehr“ hilfreich war, 36,36 % bewerteten es als „ziemlich“ hilfreich. 50 % der Befragten fand das App-basierte Training „modern und motivierend“, die andere Hälfte hätte sich jedoch mehr persönliche Betreuung gewünscht.

**Schlussfolgerung:**

Die Ergebnisse decken sich mit den Ergebnissen bereits veröffentlichter Studien: Heranwachsende sind zwar prinzipiell offen für digitale Formate, jene werden aber kaum verbindlich und kontinuierlich genutzt.

## Abstract (en)

**Background:**

The coronavirus disease 2019 (COVID-19) pandemic has had an immense impact on psychic health. Children and adolescents are considered especially vulnerable which is why health promotion and prevention programs are important and should be designed in a motivating way to be attractive to this age group. The aim of this work is to investigate whether young people can be reached with the help of innovative digital formats for health promotion.

**Methods:**

An app-based training to promote self-regulation was offered to adolescents in lower secondary education in autumn 2020. Data were collected using an app. The types of motivation to participate in the training were analyzed. In addition, the students were able to rate the attractiveness of the training with the help of a self-designed evaluation sheet.

**Results:**

Of the 91 registered participants, 39.56% completed the study. In all, 40.91% of the students stated that the training was “very” helpful and 36.36% rated it as “quite” helpful. Although 50% of the respondents found the app-based training “modern and motivating”, the other half would have preferred more personal support.

**Conclusion:**

The results confirm previous study results with health apps in children and adolescents. This age group is interested in digital formats, but health apps are not used continuously in this age group unless obligatory.

Die COVID-19-Pandemie („coronavirus disease 2019“) gilt als „Herausforderung für die psychische Gesundheit“ [1, 7, 16, 20]. 82,6 % der befragten deutschen Kinder und Jugendlichen gaben im Winter 2021 an, „äußerst belastet“ bzw. „sehr belastet“ zu sein [15]. Homeschooling, Kontaktbeschränkungen, beengte Wohnverhältnisse, existenzielle finanzielle Sorgen der Eltern – das alles sind Belastungen dieser Pandemie [3, 4, 14]. Kinder und Jugendliche benötigen also gerade jetzt Strategien zur Stressbewältigung und Selbstregulation, die schnell zu erlernen und leicht im Alltag umzusetzen sind [23]. Wie kann man diese Zielgruppe also für Gesundheitsförderung motivieren?

## Wege der digitalen Gesundheitsförderung für Kinder und Jugendliche während der COVID-19-Pandemie

Gesundheitsförderung stellt gerade in dieser Altersgruppe eine besondere Herausforderung dar [5, 6]. Eine US-amerikanische Studie belegt zwar, dass sich 84 % der befragten 13- bis 18-jährigen Jugendlichen schon mithilfe des Internets über aktuelle Gesundheitsthemen informiert haben [22]. Themen ohne direkten Gegenwartsbezug wie Prävention oder Gesundheitsförderung sind dagegen für diese Altersgruppe weniger relevant. So gab beispielsweise in einer Befragung zur Attraktivität von Programmen zur Stressbewältigung nur jeder 10. der befragten Jugendlichen an, „große Lust“ auf eine Teilnahme zu haben [6]. Psychisch belastete Schüler*innen, chronisch Kranke und in der Freizeit häufig gestresste Schüler*innen wiesen ein erhöhtes Interesse auf: Die Teilnehmer*innen müssen also das Thema für ihre eigene aktuelle Lebenswirklichkeit als wichtig bewerten.

Die Ergebnisse dieser Studie geben Hinweise darauf, dass Motivation (also die psychische Energie, die zur Initiierung und Aufrechterhaltung zielgerichteten Handelns führt) eine zentrale Voraussetzung für die Teilnahme an gesundheitsfördernden Programmen ist [21, 21]. Um diese zu steigern, sollten die entsprechenden Inhalte ansprechend und adressatengerecht präsentiert werden. Hier scheint der digitale Weg besonders gewinnbringend zu sein: Online-basierte Angebote können gerade auf diese Altersgruppe motivierend und attraktiv wirken, da sie einen hohen Grad an Unterhaltung, Information, Anonymität, Flexibilität und Innovation bieten [8, 9, 24]. Das bisherige Angebot an diesen App-Formaten für Kinder und Jugendliche ist aufgrund der permanenten dynamischen Entwicklung sehr unübersichtlich. Zudem variieren die Angebote je nach vorhandenem Endgerät. Aktuelle Studien weisen jedoch darauf hin, dass die Mehrheit der Apps nicht wissenschaftlich evaluiert wurde [8, 9].

## Forschungsfrage und Hypothesen

Aufgrund der hier dargestellten zahlreichen Vorteile von Online-Angeboten wurde ein App-basiertes Training zur Förderung der Selbstregulation für die Sekundarstufe I konzipiert und Jugendlichen angeboten.

Damit verbunden war die Fragestellung, ob diese Altersgruppe auch während der Pandemie auf diesem Weg für Gesundheitsförderung zu erreichen und für die konsequente Nutzung der App zu motivieren ist. Dabei wurden ausgehend von den oben angeführten theoretischen Erkenntnissen die folgenden Hypothesen aufgestellt:

- Akzeptanz des Trainings: Es wurde davon ausgegangen, dass die Mehrheit der Jugendlichen Interesse daran hat, dieses Angebot zu nutzen. Dabei wurde auch angenommen, dass die Schüler*innen, die sich für die Teilnahme entschieden haben, einen hohen Grad an intrinsischer Motivation besitzen. Außerdem wurde die Hypothese aufgestellt, dass Mädchen einen höheren Grad an Interesse aufweisen als Jungen und gleichzeitig auch jüngere Schüler*innen einen höheren Grad an intrinsischer Motivation als ältere Schüler*innen besitzen. Auch wurde davon ausgegangen, dass sich die Art der Motivation schulartspezifisch unterscheidet.
- Nutzungsverhalten: Es wurde angenommen, dass Schüler*innen, die das Training bis zur Evaluation I absolvieren, ein höheres Interesse am Training zeigen als Schüler*innen, die das Training vorzeitig abgebbrechen. Gleichzeitig bestand die Hypothese, dass bei Schüler*innen, die das Training vorzeitig abbrechen, die extrinsische Motivation ausgeprägter ist, als bei Schüler*innen, die das Training bis zur Evaluation I absolvieren.
- Individuelle Bewertung der Trainings: Zudem wurde davon ausgegangen, dass die Mehrheit der teilnehmenden Schüler*innen dieses App-basierte Training abschließend als hilfreich und unterstützend bewertet.

Diese vorliegende Fragestellung war Teil eines Forschungsprojekts, bei dem es auch darum ging, die Wirksamkeit verschiedener stressmindernder Strategien in Bezug auf Stressbelastung und Stressvulnerabilität zu überprüfen.

## Methoden

### Inhaltliche Gestaltung der App

**Abb. 1:**
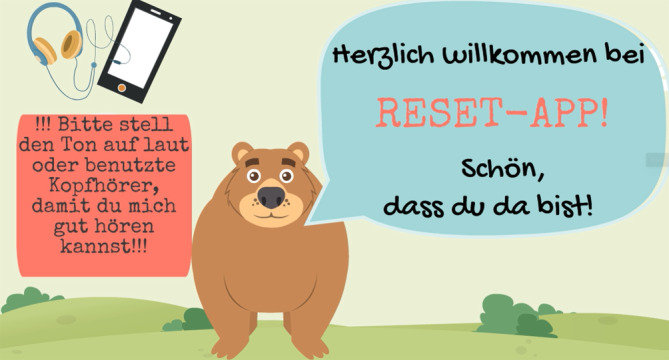
Begrüßungsbild

Es wurde ein App-basiertes Training mit einfachen und leicht umzusetzenden Methoden der Bewegung und Atemtechnik entwickelt [10]. Die App wurde „RESET-APP“ genannt, da mit ihr ein Neustart in Bezug auf das eigene negative Stresserleben gelingen soll (Abb. 1).

**Abb. 2:**
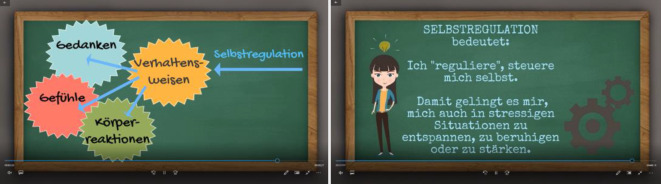
Screenshots aus dem Video „Was Stress mit uns macht“
- Selfmonitoring zur Reflexion des eigenen aktuellen negativen Stresserleben.
  Die Schüler*innen hatten nun die Aufgabe, eine Woche lang zu beobachten, in welcher Situation sie zu Stressreaktionen neigen. Sie verfassten hier jeden Tag einen kleinen Tagebucheintrag, in dem sie diese Beobachtungen in der App notierten.
- Kennenlernen selbstregulativer Strategien im Bereich Atmung und Bewegung.
  Hier lernten die Schüler*innen selbstregulative Strategien kennen, die schnell einzuüben und leicht in den Alltag zu integrieren sind. Dabei handelte es sich einerseits um das „Entschleunigte Atmen“ [12] und andererseits um bewegungsorientierte Übungen wie beispielsweise die „SURE-Methode“, also das monotone Hin-und-her-Wiegen [11].
- Erprobung der Strategien und abschließende Reflexion/Bewertung.
  Die Schüler*innen erprobten nun diese Techniken in den nächsten drei Wochen. Per Erinnerungsfunktion in der App wurden sie jeden Tag an diese Übungen erinnert. Abschließend bewerteten sie die Attraktivität des Trainings und der Übungen mithilfe eines selbst konzipierten und in die App integrierten Evaluationsbogens.

Die App bestand aus verschiedenen Modulen, welche Videos und Aufgaben beinhalteten. Die zentralen Module der App waren:

- Psychoedukation zur Stressentstehung und zu Stressreaktionen.
  Hier wurde den Schüler*innen vermittelt, wie Stress entsteht und wie er auf sie wirkt. Sie lernten dabei verschiedene schulische und soziale Stressoren kennen, erfuhren, wie diese verarbeitet werden und welche Stressreaktionen auf körperlicher, emotionaler, verhaltensbezogener und kognitiver Ebene entstehen können. Anschließend lernten sie in diesem Modul, dass es durch Techniken der Selbstregulation gelingen kann, sich in angespannten Situationen zu beruhigen und zu entspannen (Abb. 2).

Um das Training für diese Altersgruppe möglichst attraktiv zu gestalten, wurde auf eine adressatengerechte Präsentation der Inhalte geachtet. So wurden ansprechende Erklärvideos mit der Animationssoftware animaker.de entwickelt. In Bezug auf die sprachliche Darstellung wurde auch auf einfache Sprache und die Verwendung nur weniger Fachbegriffe geachtet. Als Moderator, der durch die App führt, wurde dabei ein Bär eingesetzt, der die Assoziationen „Ruhe und Gelassenheit“ hervorruft. Auch wurde beim Einsprechen der Texte auf gleichverteilte männliche und weibliche Redeanteile geachtet. Um die Identifikation mit den thematisierten stressauslösenden Situationen zu erhöhen, sprach eine Schülerin im Alter der Zielgruppe einige Textpassagen ein. Als Beispiel für Stressoren wurden typische schulische und soziale Situationen wie Konflikte im Elternhaus, unangenehme Abfragen oder angespanntes Klassenklima gewählt.

## Untersuchungsablauf und Datenanalyse

Alle Daten wurden mittels App erhoben. Direkt nach der Registrierung wurde die Motivation für die Teilnahme abgefragt. Hierzu war es möglich, 100 % auf die Motivationskomponenten (Beweggründe für die Teilnahme am Training) „Langeweile“, „Interesse“, „Eltern“ und „Freunde“ zu verteilen. Die Komponenten „Interesse“ und „Langeweile“ gehören zur intrinsischen Motivation bzw. zur Motivationsform „Selbstinitiative“ [18, 19]. „Interesse“ bedeutet ein positiver emotionaler Bezug zur Beschäftigung. Zur extrinsischen Motivation zählen die Komponenten „Eltern“ und „Freunde“ und können zur Motivationsform der fremdkontrollierten Aktivität subsummiert werden [18]: Die Aktivität – also die Teilnahme am Training – wird von anderen erwartet.

Neben dieser Motivationsabfrage konnten die Schüler*innen mittels eines selbst konzipierten Evaluationsbogens die Attraktivität und Effektivität des Trainings bewerten. Hier wurden 14 geschlossene Fragen zu den inhaltlichen Schwerpunkten sowie zur optischen und technischen Gestaltung der App gestellt. Die Schüler*innen bewerteten hier auf einer 3‑ und 5‑stufigen Skala. (*Beispielitems: Wirst du diese Übung auch in Zukunft anwenden? Würdest du einer Freundin/einem Freund das Training weiterempfehlen? Antwortmöglichkeiten ja, vielleicht, nein; Wie hilfreich war abschließend die Teilnahme am Training für dich? – Antwortmöglichkeiten sehr, ziemlich, etwas, eher nicht, überhaupt nicht*).

Ergänzend dazu fand eine weitere, nicht in die App integrierte Online-Evaluation statt, damit auch Schüler*innen, die das Training vorzeitig abbrachen, ihre persönliche Einschätzung abgeben konnten.

## Datenanalyse

Alle persönlichen Angaben der Benutzer*innen wie Anmeldedaten, Beantwortung der Fragebögen, Selbsteinschätzung im Selfmonitoring und persönliche Trainingseinschätzung wurden mit einem „Secure-hash-Algorithmus“ (SHA) anonymisiert. Zudem wurden alle Klardaten vor der Anonymisierung durch das Hinzufügen einer zufällig gewählten Zeichenfolge vor der Rückverfolgbarkeit geschützt. Die Daten wurden dann in einer SQLite-Datenbank gespeichert. Das Hosting der fertigen Applikation sowie aller damit verbundenen Daten wurde an einen professionellen Servicedienstleister übergeben. So wurde sichergestellt, dass sowohl der Quellcode als auch die generierten Daten vor Fremdzugriffen und möglichen Cyberattacken geschützt sind. Anschließend wurden Excel/CSV/Text-Dateien mit allen relevanten Informationen erzeugt und dann mithilfe der Statistiksoftware SPSS (IBM.Corp.Released 2017. IBM SPSS Statistics for Windows, Version 25.0. IBM Corp., Armonk, NY, USA) analysiert. Dieses Vorgehen wurde in der Sitzung der Ethikkommission der Universität Regensburg am 11.12.2019 genehmigt.

Zur Berechnung der geschlechter-, jahrgangsstufen- und schulartspezifischen Unterschiede in Bezug auf die Motivation wurde auf nicht-parametrische Testverfahren (Mann-Whitney-U-Test, Kruskal-Wallis-Test) zurückgegriffen, da die Voraussetzung der Normalverteilung, die eine Varianzanalyse verlangt, nicht gegeben war [13]. Auch konnten so die Unterschiede zwischen der Gruppe von Schüler*innen mit vorzeitigem Trainingsabbruch vs. der Gruppe von Schüler*innen, die das Training konsequent durchführten, ermittelt werden. Die individuelle Einschätzung zum Training von Seiten der Schüler*innen wurde deskriptiv mithilfe der Angabe von Mittelwerten und Prozentwerten dargestellt.

## Rekrutierung der Stichprobe

Auf das Projekt aufmerksam gemacht wurden insgesamt 12 bayerische Schulen (5 Gymnasien, 5 Realschulen, 2 Mittelschulen). Pandemiebedingt war es leider nicht allen angefragten Schulen möglich, das Projekt zu unterstützen. Informiert wurden mittels eines Elternrundschreibens letztendlich insgesamt 1409 Schüler*innen bayerischer weiterführender Schulen. Über die Homepage der App [17] wurden Interessierte über Studienziele, Trainingsablauf und verwendete Untersuchungsmethoden aufgeklärt. Darüber hinaus wurde eine schriftliche Einverständniserklärung der Erziehungsberechtigten eingeholt. Um die Bereitschaft zur Teilnahme zu steigern, wurde zudem darüber informiert, dass unter allen Schüler*innen, die das Training bis zum Ende absolvieren, ein iPad verlost würde.

Zeitpunkt der Datenerhebung war Mitte Oktober bis Mitte November 2020. Die Jugendlichen in Bayern nahmen zu dieser Zeit unter strengen Hygienebedingungen am Präsenzunterricht teil, wobei es oftmals vorkam, dass einige Schüler*innen oder auch ganze Schulklassen wegen COVID-19-Verdacht oder Erkrankung dem Unterricht fernbleiben mussten.

## Ergebnisse

### Stichprobenbeschreibung und Stichprobenentwicklung

**Abb. 3:**
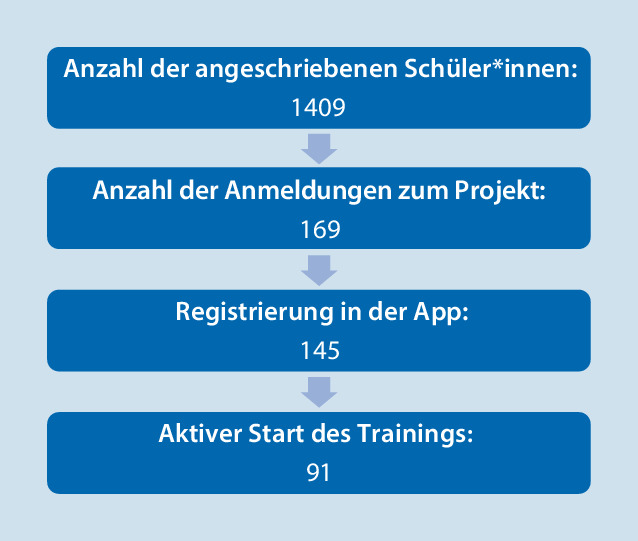
Entwicklung der Teilnehmer*innenzahl

Per E‑Mail angemeldet für diese Studie haben sich 169 Schüler*innen, registriert in der App 145. Dabei handelte es sich um 84 Jungen und 61 Mädchen. Die registrierten Jugendlichen waren zwischen 12 und 17 Jahre alt (mittleres Alter = 14,71 Jahre; SD = 10,7 Monate). 55 dieser Schüler*innen besuchten das Gymnasium, 14 die Mittelschule und 76 die Realschule. 91 Schüler*innen starteten aktiv das Training (Abb. 3). Das Training wurde von 36 Schüler*innen vollständig absolviert. Teilweise wurde auch während der Beantwortung der Fragebögen abgebrochen, was die unterschiedlichen Angaben zur Stichprobengröße in den nachfolgenden Rechenschritten erklärt.

## Motivation

Durch die Motivationsabfrage zu Beginn des Trainings wurde deutlich, welche Art von Motivation bei der Teilnahme am Training für die Schüler*innen von Bedeutung war: Die intrinsische Motivationskomponente „Interesse“ wies durchschnittlich den prozentualen Stellenwert 46,01 auf, „Langeweile“ dagegen 9,25. Der Wunsch der Eltern, dass ihre Kinder am Training teilnehmen, lag bei 34,89. Eine weniger wichtige Bedeutung spielte dagegen die Teilnahme von Freunden (Tab. 1).

**Tab. 1:** Art der Motivation in der Gesamtstichprobe

Art der Motivation in der Gesamtstichprobe – deskriptive Statistik (*n* = 91)						
–	–	*n*	Minimum	Maximum	*M*	*SD*
*Art der Motivation*	*Langeweile*	91	0,00	56,72	9,25	13,01
	*Interesse*	91	0,00	100,00	46,01	30,08
	*Eltern*	91	0,00	100,00	34,89	27,95
	*Freunde*	91	0,00	56,59	9,85	15,36

*M* Mittelwert, *SD* Standardabweichung

Es lagen keine geschlechterspezifische (*U* = 836; *z* = 1,25; *p* = 0,211), jahrgangsstufenbezogene (*H[2]* *=* 1,11; *p* = 0,574) oder schulartspezifische Unterschiede vor (*H[2]* *=* *0,*51; *p* = 0,777).

Es zeigte sich, dass diejenigen, die das Training vorzeitig abgebrochen hatten, einen geringeren Grad an „Interesse“ (*Mdn* = 33,38, *n* = 55) aufwiesen, als diejenigen, die das Training bis zum Ende absolvierten (*Mdn* = 55,45, *n* = 36). Hier lag ein statistisch signifikanter Unterschied vor (*U* = 720; z = 2,20; *p* = 0,028). Die Effektstärke nach Cohen (2013) liegt bei r = 0,23, was einem schwachen Effekt entspricht [2]. Zudem war erkennbar, dass bei denjenigen, die das Training vorzeitig abgebrochen hatten, die Motivation „Eltern“ signifikant höher ausgeprägt war (*Mdn* = 38,70) als bei denjenigen, die bis zur Evaluation I das Training absolvierten (*Mdn* = 22,90; *U* = 707; *z* = 2,29; *p* = 0,022). Die Effektstärke liegt bei r = 0,24, was einem schwachen Effekt entspricht.

## Persönliche Trainingseinschätzung

In Bezug auf die persönliche Trainingseinschätzung (*n* = 22) empfand die Mehrheit der Schüler*innen das Training als hilfreich. So gaben 40,91 % der Schüler*innen an, dass das Training „sehr“ hilfreich war, 36,36 % bewerteten es zudem als „ziemlich“ hilfreich. 18,18 % empfanden es dagegen als „kaum“ hilfreich und 4,55 % als „überhaupt nicht“ hilfreich. 86,36 % würden die App weiterempfehlen. Dass das Training per App angeboten wurde, fanden 50 % der Befragten (*n* = 31) modern und motivierend, 20 % wäre ein Kurs an der Schule lieber gewesen und für 30 % wäre eine Kombination aus App und Kurs an der Schule optimal.

## Diskussion

Gesundheits-Apps haben das Ziel, mit evidenzbasierten Methoden das physische und psychische Wohlbefinden zu fördern [8]. Daran anlehnend war es Ziel dieser Arbeit zu untersuchen, ob und wann ein App-basiertes Angebot zur Förderung der Selbstregulation und der Stressbewältigung zu Zeiten der Pandemie Jugendliche erreichen kann.

Von den 1409 angeschriebenen Schüler*innen zeigten sich nur 11,99 % prinzipiell interessiert an einer Teilnahme am Training. Aktiv das Training starteten nur 6,46 %. Das zurückhaltende Interesse an der Teilnahme am Training deckt sich mit einer Studie zur Attraktivität von Programmen zur Stressbewältigung [6]. Hierfür wurden 1699 Schüler*innen der 5. bis 10. Klasse weiterführender Schulen im Schuljahr 2001/2002 befragt. 9,9 % der Befragten gaben an, „große Lust“ zur Teilnahme an einem Stressbewältigungstraining zu haben, 25,3 % hatten „eher große Lust“, während 36,3 % „eher keine Lust“ und 28,4 % „keine Lust“ angaben.

Die Tatsache, dass das Angebot der vorliegenden App so wenig genutzt wurde, kann daran gelegen haben, dass sowohl Eltern also auch ihre Kinder durch Distanzlernen und Homeschooling bereits digital übersättigt waren. Für die konkrete Motivation für die Anmeldung und Teilnahme am Training wirken also, neben überdauernden motivationalen Tendenzen, mit Sicherheit auch aktuelle situative Merkmale wie die COVID-19-Pandemie oder der damit verbundene Lockdown [19]. Die hier konzipierte RESET-APP könnte demnach u. U. in einem normal ablaufenden Schulalltag motivierender und innovativer wirken.

Es bestätigte sich, dass die Art der Motivation maßgeblich das Verhalten während des Trainings beeinflusste. So wiesen Schüler*innen, die das Training bis zum Ende absolvierten, einen höheren Grad an Interesse auf als Schüler*innen, die das Training vorzeitig abbrachen. Gleichzeitig gaben Schüler*innen, die das Training vorzeitig abbrachen, ausgeprägter die Eltern als Grund für die Teilnahme an als Schüler*innen, die das Training bis zum Ende absolvierten. Hier wird also die entscheidende Bedeutung intrinsischer Motivation in Zusammenhang mit der Wertkomponente „Interesse“ für den Trainingserfolg und die Trainingsteilnahme deutlich: Intrinsische Motivation führt zu einer Wertschätzung des Gegenstands und der damit verbundenen Wissenserweiterung, in dem Fall die Möglichkeit, durch die App neue Strategien der Selbstregulation kennenzulernen [18, 19].

Die Mehrheit von denjenigen, die das Training bis zum Ende absolviert hatten, empfand das Training als „sehr“ bzw. „ziemlich“ hilfreich und würde es auch weiterempfehlen. Dass das Training per App angeboten wurde, fanden 50 % der Befragten (*n* = 31) modern und motivierend, 20 % wäre ein Kurs an der Schule lieber gewesen und für 30 % wäre eine Kombination aus App und Kurs an der Schule optimal.

Auch wenn diese vorliegenden Ergebnisse dafürsprechen, dass Jugendliche Gesundheits-Apps – wie vorher angenommen – prinzipiell als attraktiv und hilfreich bewerten, absolvierten nur 39,56 % der Schüler*innen das Training bis zum Ende: Die konsequente Nutzung digitaler Formate ist also ein limitierender Faktor. Eine US-amerikanischen Studie unter 13- bis 18-Jährigen [9, 24] kam zu einem ähnlichen Ergebnis [24]: 47 % der Jugendlichen, die eine Gesundheits-App heruntergeladen hatten, gaben an, die App dann tatsächlich kaum zu benutzen, nur 8 % benutzten sie konsequent. Auch wenn also die generelle Bereitschaft zur Arbeit mit der App vorhanden ist, scheint es für diese Altersgruppe schwierig zu sein, auch langfristig ihre Motivation aufrecht zu erhalten. Hier fehlen u. U. persönliche Betreuung und Unterstützung.

Auch scheinen technische Schwierigkeiten besonders zu demotivieren [23]. Da die vorliegende App im Rahmen eines Forschungsprojekts konzipiert wurde, waren die Ressourcen dafür begrenzt. Auch wenn die Schüler*innen bei technischen Schwierigkeiten per E‑Mail betreut wurden, ist zu vermuten, dass einige Schüler*innen diesbezüglich über wenig Frustrationstoleranz verfügten.

Zusammenfassend ist also zu sagen, dass gesundheitsbezogene Apps generell motivierend und innovativ auf Jugendliche wirken [8]. Die konsequente Nutzung dieser setzt jedoch einen hohen Grad an intrinsischer Motivation, Selbstständigkeit und Disziplin voraus. Auch wenn bei der Konzeption der App auf eine ansprechende und altersgerechte Gestaltung geachtet wurde, konnte nicht verhindert werden, dass nur ein geringer Anteil der registrierten Schüler*innen das Training bis zum Ende absolvierte. Um die Attraktivität der App zu steigern, sollten bei einer Überarbeitung mehr spielerische, und damit unterhaltsame, Elemente in die App integriert werden [22]. Durch den Einsatz von „gamifications“ könnten so beispielsweise Belohnungssysteme wie das Erreichen von Leveln oder das Sammeln von Punkten eingeführt werden, was mit Sicherheit die Motivation für die konsequente Bearbeitung steigern würde.

Daneben scheint gerade im sensiblen schulpsychologischen Bereich eine persönliche Betreuung bzw. Ansprache wichtig zu sein, um die Akzeptanz zu erreichen und die Relevanz der entsprechenden Themen für diese Altersgruppe zu betonen [23].

## Limitation

Um die Aussagekraft der Untersuchungsergebnisse zu erhöhen, wären u. a. folgende Änderungen hinsichtlich des Studiendesigns notwendig:

- Vergrößerung der Stichprobe, damit verbunden auch eine Gleichverteilung in Bezug auf Geschlecht, Schulart und Alter,
- Ausweitung des Altersspektrums,
- erneute Erhebung in einem normalen Schullalltag, um die Attraktivität des der App nochmals zu überprüfen,
- Einsatz von „gamification“, um die Motivation der Jugendlichen zu steigern [22],
- regelmäßige, persönliche Betreuung während des Trainings.

## Fazit für die Praxis

- Präventionsmaßnahmen im psychologischen Bereich für die Altersgruppe der Jugendlichen stellen eine große Herausforderung dar. Die Darbietung sollte auf moderne, innovative und anschauliche Art und Weise erfolgen.
- Apps können dieser Forderung Rechnung tragen, jedoch fehlt hier einigen Schüler*innen der persönliche Kontakt. Eine Kombination aus App-basierten bzw. digitalen Formaten wie ansprechenden Videosequenzen oder digitalen Arbeitsaufträgen und persönlicher Anleitung scheint am effektivsten zu sein.
